# Radiographic Analysis on the Distortion of the Anatomy of First Metatarsal Head in Dorsoplantar Projection

**DOI:** 10.3390/diagnostics10080552

**Published:** 2020-08-02

**Authors:** Jessica Grande-del-Arco, Ricardo Becerro-de-Bengoa-Vallejo, Patricia Palomo-López, Daniel López-López, César Calvo-Lobo, Eduardo Pérez-Boal, Marta Elena Losa-Iglesias, Carlos Martin-Villa, David Rodriguez-Sanz

**Affiliations:** 1 Facultad de Enfermería, Fisioterapia y Podología, Universidad Complutense de Madrid, 28040 Madrid, Spain; jessicagrandedelarco@gmail.com (J.G.-d.-A.); ribebeva@ucm.es (R.B.-d.-B.-V.); cescalvo@ucm.es (C.C.-L.); perez.boal@gmail.com (E.P.-B.); podologiamartinvilla@gmail.com (C.M.-V.); davidrodriguezsanz@ucm.es (D.R.-S.); 2 University Center of Plasencia, Universidad de Extremadura, 10600 Plasencia, Spain; patibiom@unex.es; 3 Research, Health and Podiatry Group, Department of Health Sciences, Faculty of Nursing and Podiatry, Universidade da Coruña, 15403 Ferrol, Spain; 4 Faculty of Health Sciences, Universidad Rey Juan Carlos, 28922 Alcorcón, Spain; marta.losa@urjc.es

**Keywords:** First metatarsal head, foot, radiological health, metatarsal bones

## Abstract

**Background:** The diagnostic of flat and crest-shaped of first metatarsal heads has been associated as an important risk factor for hallux deformities, such as hallux valgus and hallux rigidus. The rounded form of the first metatarsal head on the dorsoplantar radiograph of the foot has been believed to be associated with the development of hallux valgus. **Purpose:** The aim of this study was to clarify the effect of tube angulation on the distortion of first metatarsal head shape, and verify the real shape of the metatarsal head in anatomical dissection after an X-ray has been taken. **Materials and Methods:** In this prospective study at Universidad Complutense de Madrid, from December 2016 to June 2019, 103 feet from embalmed cadavers were included. We performed dorsoplantar radiograph tube angulation from 0° until 30° every 5° on all specimens; then, two observers verified the shape of the first metatarsal head in the radiographs and after its anatomic dissection. Kappa statistics and McNemar Bowker tests were used to assess and test for intra and interobserver agreement of metatarsal shape. **Results:** We calculated the intraobserver agreement, and the results showed that the first metatarsal head is distorted and crested only when the angle of the X-ray beam is at 20° of inclination (*p* < 0.001). The interobserver agreement showed good agreement at 0°, 5°, 10°, 20°, and 25° and was excellent at 30° (*p* < 0.001). **Conclusion:** All of the studies that we identified in the literature state that there are three types of shapes of the first metatarsal head and relate each type of head to the diagnosis of a foot pathology, such as hallux valgus or hallux rigidus. This study demonstrates that there is only the round-shaped form, and not three types of metatarsal head shape. Therefore, no diagnoses related to the shape of the first metatarsal head can be made.

## 1. Introduction

Hallux valgus (HV) is a highly prevalent foot deformity estimated to affect 23% of adults and 35.7% of elderly individuals (1). HV presents a significant individual and public health burden, due to the high occurrence of related orthopedic foot surgery [1], and its association with foot pain [2,3], osteoarthritis (OA) at the first metatarsophalangeal joint (MTPJ), impaired gait patterns [4], poorly coordinated stability and an increased risk of falls in older adults [5,6].

While the development of HV is believed to be multifactorial, the exact etiology remains unclear [7]. Previous studies have suggested that several structural factors might be characteristic of HV, including various radiographic angles, first MTPJ congruency, metatarsal length, metatarsal head shape, sesamoid position, first metatarsocuneiform joint flexibility, and pes planus [8,9].

First metatarsal head shape has been routinely assessed by orthopedic surgeons radiographically, and has been addressed by as many as 24 authors, as well as in systematic reviews [7] to claim that shape is significant in the development of HV, and it has been classified as three types: round, square and crest [8,10,11,12,13,14,15,16,17,18,19,20,21,22,23,24,25,26,27,28], with the crest type being the most stable to prevent the development of HV and the round shape contributing to the development of HV, and it is one of the factors in recurrence after hallux valgus surgery [11,14,15,17,18,19,21,22,23,28,29,30,31,32].

Several authors have reported a relationship between a round-shaped metatarsal head and hallux valgus, but have not detected a strong correlation due to a lack of substantial data between them. Therefore, it is unknown whether a metatarsal head shape predisposes one to the development of hallux valgus.

In patients with HV, radiographs are obtained as part of a clinical evaluation. On these radiographs, angular measurement is used to determine the severity of deformation. A 1951 study [33] analyzed sources of error in the production and measurement of radiographs of the foot. This publication illustrated the need for the standardization of the radiograph of the dorsoplantar view of the foot, which has been widely advocated [33,34].

Despite this, various authors who described “their” standard technique of the dorsoplantar radiograph use a craniocaudal tube angulation of 5° [35], 15° [36,37,38], or 20° [39], but The American Orthopaedic Foot and Ankle Society recommended a tube angulation of 15° [40].

One study has been performed with a tube angulation of 20° in patients with HV, and states a relatively small reduction in the distortion of the intermetatarsal angles, but did not evaluate other anatomical structures.

To our knowledge, a systematic analysis of the relationship between tube angulation and the distortion due to the projection of the actual anatomy on the radiographs has not been performed beyond 20°.

The goal of this study was to analyze the effects and distortion that occur in the shape of the first metatarsal head when performing a dorsoplantar X-ray with the angled X-ray tube from 0° to 30° in anatomical specimens, and subsequently performing its dissection, to determine if the anatomic and radiographic findings correlate.

## 2. Material and Methods

From December 2016 to June 2019, 173 feet from embalmed cadavers were included in the study from Donation Center of the Bodies and Dissection Rooms of the Complutense, The University of Madrid. The institutional review board of the Rey Juan Carlos University approved with data 14 february of 2017 the study under number 27122011600917.

Those samples that included the complete foot with the distal third of the tibia and those samples that clinically showed no signs of surgical intervention were included in the selection of anatomical pieces.

The inclusion criteria followed in the radiographic evaluation were adult feet with radiographic images, in which all the growth cartilages of the foot and the distal third of the tibia and fibula were completely closed. It was required that the radiographic images showed the entire foot. Radiographs that showed traumatic or degenerative changes of the sesamoids or the surface of the first MTP joint, the presence of hallux valgus, or an intermetatarsal angle greater than 12° were excluded, as established in the article by Durrant et al. [41]

Each specimen was clinically examined to determine if they presented any deformity and those anatomical pieces that presented deformities in the foot, such as hallux valgus, hallux rigidus, osteoarthritis in the first MTF joint, fractures in the first metatarsal or presence of implants, patients with obviously abnormal shapes of the first metatarsal, due to fracture, invasion of the tumor, or congenital disease, were excluded.

Because of this, only 103 complying with the inclusion criteria were used outlined in the study. 

The variables to be studied on the radiographs were the shape of the head of the 1st metatarsal, establishing the following categories: Round, square and “with crest”, as reported in the literature [8,10,11,12,13,14,15,16,17,18,19,20,21,22,23,24,25,26,27,28].

### 2.1. Radiographic Protocol

The optimal tube angulation was defined as the angulation that was associated with the smallest average distortion. Besides the varying tube angulation, the geometry of this projection was identical to the standard technique of a dorsoplantar radiograph.

An Optima Xr200amx portable radiology equipment from Ge Heticare, 30 kW (GE HEALTHCARE, Madrid, Spain www.gehealthcare.com) was used with a 24 × 30 cm chassis and FireCR Spark Medical digital reader, 4dmedical, Valencia, Madrid.

The anatomical feet were placed on the radiographic plate in a neutral position, taking into account the methodology and protocol proposed by the studies by Venning and Hardy (1951) and Tanaka, Takakura, Kumai, Samotoy Tamai (1995) [33,42].

The standard dorsal, plantar radiographic projection proposed by several researchers was used: the X-ray beam tilts at 15° at a distance of 100 cm, to ensure the accuracy of these records are obtained from various articles [10,33,42].

### 2.2. Radiographic Representation

The samples underwent several images of the first metatarsal at different degrees of the beam projector. We perform a radiographic analysis with different degrees of projection.

We used a variable craniocaudal tube angulation in a sagittal plane 0°, 5°, 10°, 15°, 20°, 25°, and 30°, and the beam direction was set parallel to the axis of the foot and centered on the second metatarsal tarsus [42].

**Figure 1:**
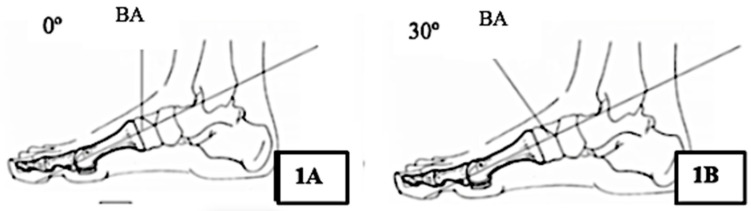
Position of the X-ray beam at a distance of 100 cm to obtain precision in the images. Abbreviations: BA: Beam angle. (**A**) relationship between the angulations with an X-ray beam projection at 0°; (**B**) Relationship between the angulations with an X-ray beam projection at 30°.

During X-ray imaging, the X-ray beam is perpendicular to the image intensifier, and the foot is positioned parallel to the image intensifier (Figure 1).

A neutral position, with 0° of inclination and rotation, avoiding pronation or supination of feet and beam direction focused on the second wedge joint as an exponent [43].

### 2.3. X-Ray Observation

The shape of the head of the first metatarsal was classified into three types, according to several authors: round, flat, and with crest [8,10,11,12,13,14,15,16,17,18,19,20,21,22,23,24,25,26,27,28]. The observation consisted of the two assessors measuring relevant measures of 103 randomly chosen feet radiographs, and then 1 week later re-measuring all radiographs without reference to previous results.

After observing the radiographs, the samples were dissected to assess the shape of the first metatarsal head by the same two observers who assessed the radiographs.

### 2.4. Statistical Analysis

Kappa statistics and generalized McNemar tests were used to assess and test for agreement. The shape of the first metatarsal head was polycotomized into three groups; “round”, “flat”, and “crest”. As suggested by Landis and Koch, we interpreted the kappa values as follows: <0.20 indicates poor agreement, 0.21–0.40 fair, 0.41–0.60 moderate, 0.61–0.80 good agreement, and >0.80 indicates excellent agreement [44].

The McNemar Bowker test describes whether the marginal distributions of two measures are similar, as one would expect if the measures agree.

Data were analyzed using IBM SPSS Statistics, version 22 statistical software (SPSS Inc, Chicago, IL, USA). Statistical significance was set at *p* < 0.05, and Confidence Interval (IC) to 95%.

## 3. Results

The interobserver agreement by Kappa analysis (Table 1) showed a moderate agreement at 15°, good agreement at 0°, 5°, 10°, 20° and 25° and was excellent at 30°.

To calculate intraobserver agreement, results were compared against angle beams. Table 2 shows the intraobserver A agreement regarding when the first metatarsal head gets distorted and appears crested. Results indicate that this occurs when the angle of the X-ray beam is at 20° of inclination. These results are similar for intraobserver B (Table 3), where the distortion of the same head occurs at 20° relative to 15° (*p* < 0.001).

Finally, after dissecting the 103 anatomical specimens, we found that all the first metatarsal heads had a round shape and none with a square shape or a crested head, showing perfect intra and interobserver agreement. 

## 4. Discussion

The purpose of this study was to determine the presence of a distortion effect in the first metatarsal shape, due to the angulation of the X-ray beam.

Most articles on the measurement of dorsoplantar radiographs report a 15° [33,42] or 20° craniocaudal tube angulation. The American Orthopaedic Foot and Ankle Society has recommended a tube angulation of 15° [40].

We used observations of radiographs in this study. This technique used a craniocaudal tube angulation in a sagittal plane 0°, 5°, 10°, 15°, 20°, 25° and 30°, to evaluate the shape of the first metatarsal and including dissection of 103 feet embalmed cadaver by both observers.

We focused on the distorting effects of the tube angulation in the shape of the first metatarsal. We found that the distortion of the shape of the first metatarsal was minimal when the radiograph was made without angulation, or the beam angle was less than 20°.

**Figure 2:**
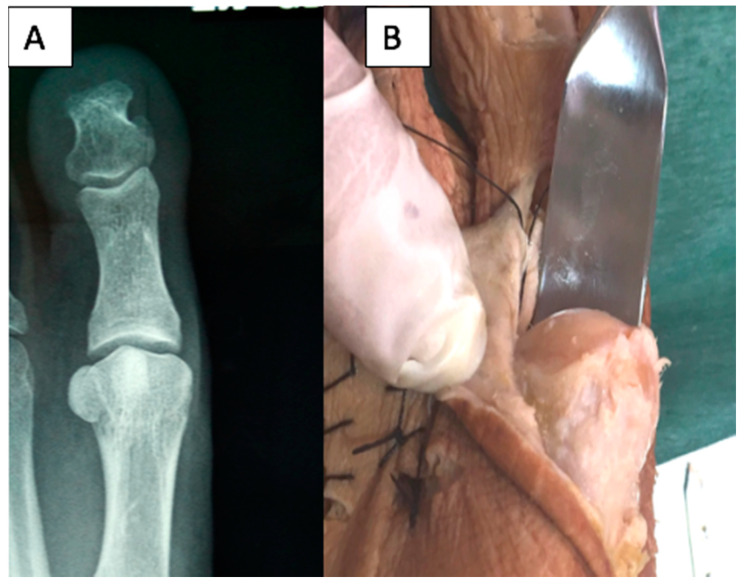
Views of a first metatarsal head showing distortion to appear crest shaped in a radiographic image performed to 30° (**A**) and after dissection revealing a round shape (**B**).

Both observers agree that the shape of the metatarsal head is distorted in projections in which the X-ray beam with angulations is equal to or greater than 20° (Figure 2).

In this study, an association between a flat- or crested-shaped head of the first metatarsal with pathologies, such as hallux rigidus or hallux limitus, cannot be supported, because these shapes are the result of distortion caused by tube angulation.

Another reason for the distortion of the first metatarsal head with the X-ray beam correctly positions at 15° is that the normal first metatarsal declination angle is 21° angle between the axis of the first metatarsal and a horizontal linear [45], and in the flat foot, the first metatarsal declination angle is lower.

So, we postulated that when tube angulation in a sagittal plane is 15° [33,42] in a normal foot with a first metatarsal angle declination of 21°, the possibility of deformation or distortion of the first metatarsal head is minimized.

Instead, if the first metatarsal bone is dorsiflexed as a flat foot, the first metatarsal angle declination is lower, and the angle between the X-ray beam and the axis of the first metatarsal bone is a higher, thus maximizing distortion of the first metatarsal head.

In light of these findings, it seems necessary to control the beam angulation to 5–10° in dorsoplantar X-rays of the flat loading foot, to avoid the presence of the crested or flat shape, which are artifacts produced by the angulation of the tube.

## 5. Conclusions

All of the articles that we identified state that there are three types of shapes of the first metatarsal head, and all authors relate each type of head to the diagnosis of a foot pathology, such as hallux valgus or hallux rigidus. This study demonstrates that there is only a round shape, and not three types of metatarsal head shape, and therefore, no diagnoses related to the shape of the first metatarsal head can be made.

A clinician should be aware that, in patients with flat feet, dorsoplantar with weight projection should be taken at an angle of the 5 to 10° beam.

## Figures and Tables

**Table 1:** Interobserver agreement about shape first metatarsal head in beam angle 0–30°.

Reader B							
	Shape Metatarsal Head	Beam Angle	Round F (%)	Flat F (%)	Crest F (%)	Total F (%)	Kappa (*p*)
**Reader A**	0°	Round	69 (67.0%)	10 (9.7%)	0 (0.0%)	79 (76.7%)	0.679 (<0.001)
		Flat	3 (29%)	21 (20.4%)	0 (0.0%)	24 (23.3%)	
		Crest	0 (0.0%)	0 (0.0%)	0 (0.0%)	0 (0.0%)	
		Total F (%)	72 (69.9%)	32 (30.1%)	0 (0.0%)	103 (100%)	
	5°	Round	72 (69.9%)	9 (8.7%)	0 (0.0%)	81 (78.6%)	0.715 (<0.001)
		Flat	2 (1.9%)	20 (19.4%)	0 (0.0%)	22 (21.4%)	
		Crest	0 (0.0%)	0 (0.0%)	0 (0.0%)	0 (0.0%)	
		Total F (%)	74 (71.8%)	29 (28.2%)	0 (0.0%)	103 (100%)	
	10°	Round	73 (70.9%)	12 (11.7%)	0 (0.0%)	85 (82.5%)	0.613 (<0.001)
		Flat	2 (1.9%)	16 (15.5%)	0 (0.0%)	18 (17.5%)	
		Crest	0 (0.0%)	0 (0.0%)	0 (0.0%)	0 (0.0%)	
		Total F (%)	75 (72.8%)	28 (27.2%)	0 (0.0%)	103 (100%)	
	15°	Round	70 (68.0%)	10 (9.7%)	0 (0.0%)	80 (77.7%)	0.548 (<0.001)
		Flat	6 (5.8%)	16 (15.5%)	0 (0.0%)	22 (21.4%)	
		Crest	1 (0.0%)	0 (0.0%)	0 (0.0%)	1 (1.0%)	
		Total F (%)	75 (72.8%)	26 (27.2%)	0 (0.0%)	103 (100%)	
	20°	Round	38 (36.9%)	7 (6.8%)	2 (1.9%)	47 (45.6%)	0.726 (<0.001)
		Flat	1 (1.0%)	12 (11.7%)	0 (0.0%)	13 (12.6%)	
		Crest	2 (1.9%)	6 (5.8%)	35 (34.0%)	43 (42.7%)	
		Total F (%)	41 (39.8%)	25 (24.3%)	37 (35.9%)	103 (100%)	
	25°	Round	6 (5.8%)	1 (1.0%)	1 (1.0%)	8 (7.8%)	0.616 (<0.001)
		Flat	0 (0.0%)	3 (2.9%)	0 (0.0%)	3 (2.9%)	
		Crest	4 (3.9%)	4 (3.9%)	84 (81.6%)	92 (89.3%)	
		Total F (%)	10 (9.7%)	8 (7.8%)	85 (82.5%)	103 (100%)	
	30°	Round	2 (1.9%)	0 (0.0%)	0 (0.0%)	2 (1.9%)	0.853 (<0.001)
		Flat	0 (0.0%)	1 (1.0%)	0 (0.0%)	1 (1.0%)	
		Crest	0 (0.0%)	1 (1.0%)	99 (96.1%)	100 (97.1%)	
		Total F (%)	2 (1.9%)	2 (1.9%)	99 (96.1%)	103 (100%)	

Abbreviations: F, frequency.

**Table 2:** Intraobserver A agreement about shape first metatarsal head in beam angle: 0° vs. 5°, 5° vs. 10°, 10° vs. 15, 15 vs. 20°, 20 vs. 25°, 25° vs. 30°.

Reader A							
	Shape Metatarsal Head	Beam Angle	Round F (%)	Flat F (%)	Crest F (%)	Total F (%)	Mcnemar (*p* *)
**Reader A**	0° vs. 5°	Round	79 (76.7)	0 (0.0%)	0 (0.0%)	79 (76.7%)	2 (0.157)
		Flat	2 (1.9%)	22 (21.4%)	0 (0.0%)	24 (23.3%)	
		Crest	0 (0.0%)	0 (0.0%)	0 (0.0%)	0 (0.0%)	
		Total F (%)	81 (78.6%)	22 (21.4%)	0 (0.0%)	103 (100%)	
	5° vs. 10°	Round	80 (77.7%)	1 (1.0%)	0 (0.0%)	81 (78.6%)	2.667 (0.102)
		Flat	5 (4.9%)	17 (16.5%)	0 (0.0%)	22 (21.4%)	
		Crest	0 (0.0%)	0 (0.0%)	0 (0.0%)	0 (0.0%)	
		Total F (%)	85 (82.5%)	18 (17.5%)	0 (0.0%)	103 (100%)	
	10° vs. 15°	Round	79 (76.7%)	5 (4.9%)	1 (0.0%)	85 (82.5%)	3.667 (0.160)
		Flat	1 (1.0%)	17 (16.5%)	0 (0.0%)	18 (17.5%)	
		Crest	1 (0.0%)	0 (0.0%)	0 (0.0%)	0 (0.0%)	
		Total F (%)	80 (77.7%)	22 (21.4%)	1 (1.0%)	103 (100%)	
	15° vs. 20°	Round	47 (45.6%)	1 (1.0%)	32 (31.1%)	80 (77.7%)	43 (<0.001)
		Flat	0 (0.0%)	12 (11.7%)	10 (9.7%)	22 (21.4%)	
		Crest	0 (0.0%)	0 (0.0%)	1 (1.0%)	1 (1.0%)	
		Total F (%)	47 (45.6%)	13 (12.6%)	43 (41.7%)	103 (100%)	
	20° vs. 25°	Round	7 (6.8%)	0 (0.0%)	40 (38.8%)	47 (45.6%)	50 (<0.001)
		Flat	1 (1.0%)	3 (2.9%)	9 (8.7%)	13 (12.6%)	
		Crest	0 (0.0%)	0 (0.0%)	43 (41.7%)	43 (41.7%)	
		Total F (%)	8 (7.8%)	3 (2.9%)	92 (89.3%)	103 (100%)	
	25° vs. 30°	Round	2 (1.9%)	0 (0.0%)	6 (5.8%)	8 (7.8%)	7 (0.030)
		Flat	0 (0.0%)	0 (0.0%)	3 (2.9%)	3 (2.9%)	
		Crest	0 (0.0%)	1 (1.0%)	91 (88.3%)	92 (89.3%)	
		Total F (%)	2 (1.9%)	1 (1.0%)	100 (97.5%)	103 (100%)	

Abbreviations: F: frequency. * The McNemar Bowker test was used to compare the relative prevalence of the different grades and is given as *p*-values.

**Table 3:** Intraobserver B agreement about shape first metatarsal head in beam angle: 0° vs. 5°, 5° vs. 10°, 10° vs. 15, 15 vs. 20°, 20 vs. 25°, 25° vs. 30°.

Reader B							
	Shape Metatarsal head	Beam Angle	Round F (%)	Flat F (%)	Crest F (%)	Total F (%)	Mcnemar (*p* *)
**Reader B**	0° vs. 5°	Round	72 (69.9%)	0 (0.0%)	0 (0.0%)	72 (69.9%)	2 (0.157)
		Flat	2 (1.9%)	29 (28.2%)	0 (0.0%)	31 (30.1%)	
		Crest	0 (0.0%)	0 (0.0%)	0 (0.0%)	0 (0.0%)	
		Total F (%)	74 (71.8%)	29 (28.2%)	0 (0.0%)	103 (100%)	
	5° vs. 10°	Round	74 (71.8%)	0 (0.0%)	0 (0.0%)	74 (71.8%)	1 (0.317)
		Flat	1 (1.0%)	28 (27.2%)	0 (0.0%)	29 (28.2%)	
		Crest	0 (0.0%)	0 (0.0%)	0 (0.0%)	0 (0.0%)	
		Total F (%)	75 (72.8%)	28 (27.2%)	0 (0.0%)	103 (100%)	
	10° vs. 15°	Round	73 (70.9%)	2 (1.9%)	0 (0.0%)	75 (72.8%)	0.667 (0.414)
		Flat	4 (3.9%)	24 (23.3%)	0 (0.0%)	28 (27.2%)	
		Crest	0 (0.0%)	0 (0.0%)	0 (0.0%)	1 (0.0%)	
		Total F (%)	77 (74.8%)	26 (25.2%)	0 (0.0%)	103 (100%)	
	15° vs. 20°	Round	41 (39.8%)	3 (2.9%)	33 (32.0%)	77 (74.8%)	39 (<0.001)
		Flat	0 (0.0%)	22 (21.4%)	4 (3.9%)	26 (25.2%)	
		Crest	0 (0.0%)	0 (0.0%)	0 (0.0%)	0 (0.0%)	
		Total F (%)	41 (39.8%)	25 (24.3%)	37 (35.9%)	103 (100%)	
	20° vs. 25°	Round	10 (9.7%)	4 (3.9%)	27 (26.2%)	41 (39.8%)	52 (<0.001)
		Flat	0 (0.0%)	4 (3.9%)	21 (20.4%)	25 (24.3%)	
		Crest	0 (0.0%)	0 (0.0%)	37 (35.9%)	37 (35.9%)	
		Total F (%)	10 (9.7%)	8 (7.8%)	85 (82.5%)	103 (100%)	
	25° vs. 30°	Round	2 (1.9%)	0 (0.0%)	8 (7.8%)	10 (9.7%)	14 (<0.001)
		Flat	0 (0.0%)	2 (1.9%)	6 (5.8%)	8 (7.8%)	
		Crest	0 (0.0%)	0 (0.0%)	85 (82.5%)	85 (82.5%)	
		Total F (%)	2 (1.9%)	2 (1.9%)	99 (96.1%)	103 (100%)	

Abbreviations: F, frequency. * The McNemar Bowker test was used to compare the relative prevalence of the different grades and is given as *p*-values.
